# ANCA-associated vasculitis and the impact of diffuse alveolar hemorrhage in elderly patients: a retrospective cohort study

**DOI:** 10.1007/s00296-025-05812-8

**Published:** 2025-03-03

**Authors:** Matthias Schaier, Florian Kälble, Louise Benning, Paula Reichel, Christoph Mahler, Christian Nusshag, Jonas Rusnak, Tobias Gutting, Michael Preusch, Martin Zeier, Christian Morath, Claudius Speer

**Affiliations:** 1 Department of Nephrology, Heidelberg University, INF 162, 69120 Heidelberg, Germany; 2 Department of Internal Medicine III (Cardiology, Angiology, and Pneumology), Heidelberg University, Heidelberg, Germany

**Keywords:** Anti-neutrophil cytoplasmic antibody-associated vasculitis, Infections, Elderly, Immunosuppression, Adverse drug event

## Abstract

The ANCA-associated vasculitis (AAV) has an exceptionally high morbidity and mortality especially in patients with diffuse alveolar hemorrhage (DAH). Data on DAH in elderly AAV patients is still very limited. To investigate the impact of DAH on patient survival, relapse-free survival, death from infectious complications, and the incidence of pneumonia in one of the most vulnerable but often underrepresented AAV subpopulation—elderly patients. We included 139 AAV patients in this retrospective cohort study and performed a 5-year follow-up. AAV patients were divided into patients ≤ 65 and > 65 years (“elderly”). Elderly AAV patients were further subdivided into patients with and without DAH. Relapse-free survival was comparable (*P* = 0.49) whereas overall patient survival (*P* = 0.01) was significantly lower in patients > 65 as compared to ≤ 65 years. Death due to infectious complications occurred more frequently in the elderly cohort (log-rank *P* = 0.02). Especially the incidence of pneumonia (including opportunistic pathogens) was considerably higher in elderly AAV patients (log-rank *P* = 0.001). Overall survival in elderly patients was significantly lower in patients with as compared to patients without DAH [8/18 (44%) versus 9/52 (17%) deaths (*P* = 0.02)] while relapse-free survival was again comparable (*P* = 0.87) between both groups. Notably, 6 out of 8 fatal outcomes in elderly DAH patients were associated with severe infections. In multivariate analyses, age and glucocorticoid (GC) dose at 3 months were the only predictors of death from infectious complications, whereas this could not be independently demonstrated for DAH. Life-threatening infections with (opportunistic) pneumonia are common in elderly AAV patients with DAH during the first 12 months and higher GC dose was an independent predictor of death from infectious complications.

**Supplementary Information:**

The online version contains supplementary material available at 10.1007/s00296-025-05812-8.

## Introduction

The anti-neutrophil cytoplasmic antibody (ANCA)-associated vasculitis (AAV) is an orphan disease comprising microscopic polyangiitis (MPA), granulomatosis with polyangiitis (GPA), and eosinophilic granulomatosis with polyangiitis (EGPA). MPA and GPA are characterized by small vessel inflammation that can occur in almost all organs, although kidneys and lungs are most frequently affected [1, 2]. Because the morbidity and mortality of untreated AAV is very high, a timely and broad induction therapy is essential to improve patient survival and preserve organ function [3, 4]. However, improvements in immunosuppressive treatment regimens have been offset by an unfavorable side effect profile including cardiovascular complications, an increased incidence of malignancies and, most notably, infectious complications [5]. In addition to the impact of the underlying AAV disease activity, infections contribute significantly to morbidity and are the primary cause of mortality in the first year following disease onset [6, 7].

The highest incidence of AAV is observed in individuals aged 60 and above, which classifies AAV as a disease of the elderly [8]. In addition, a study by Bloom et al. showed that age at diagnosis is associated with particular clinical features in AAV [9]. In particular, the Vascular Damage Index (VDI) increased with age at diagnosis, reflecting non-disease-specific damage characteristics and highlighting the vulnerability of these individuals [9]. Nevertheless, randomized controlled trials frequently excluded patients over the age of 70–80 years from participation. Consequently, findings on different treatment regimens, outcome parameters, and treatment-associated toxicities are predominantly based on observational studies [10]. Given the heightened susceptibility of older AAV patients to the adverse effects of immunosuppressive therapy, it is of paramount importance to tread a delicate balance between disease control and the prevention of serious infectious complications in this patient population. However, despite the considerable disease burden and treatment-related complications, older AAV patients also appear to have a relatively favorable outcome after the first year, underscoring the necessity and efficacy of adequate immunosuppression in this cohort as well [11].

Diffuse alveolar hemorrhage (DAH) is one of the most serious complications of AAV, frequently resulting in respiratory failure, necessitating intensive care and invasive ventilation [12]. Intensified immunosuppressive treatment regimens were therefore used, including e.g. the use of plasma exchange, although their efficacy has not yet been conclusively clarified [13]. Especially in elderly AAV patients with DAH the available data on treatment, outcomes, and complications are still very limited.

The objective of this study was to examine the impact of DAH on disease progression, mortality, and severe infectious complications in one of the most vulnerable but frequently encountered and previously underrepresented AAV subpopulations—elderly AAV patients.

## Methods

### Patient population

**Fig. 1:**
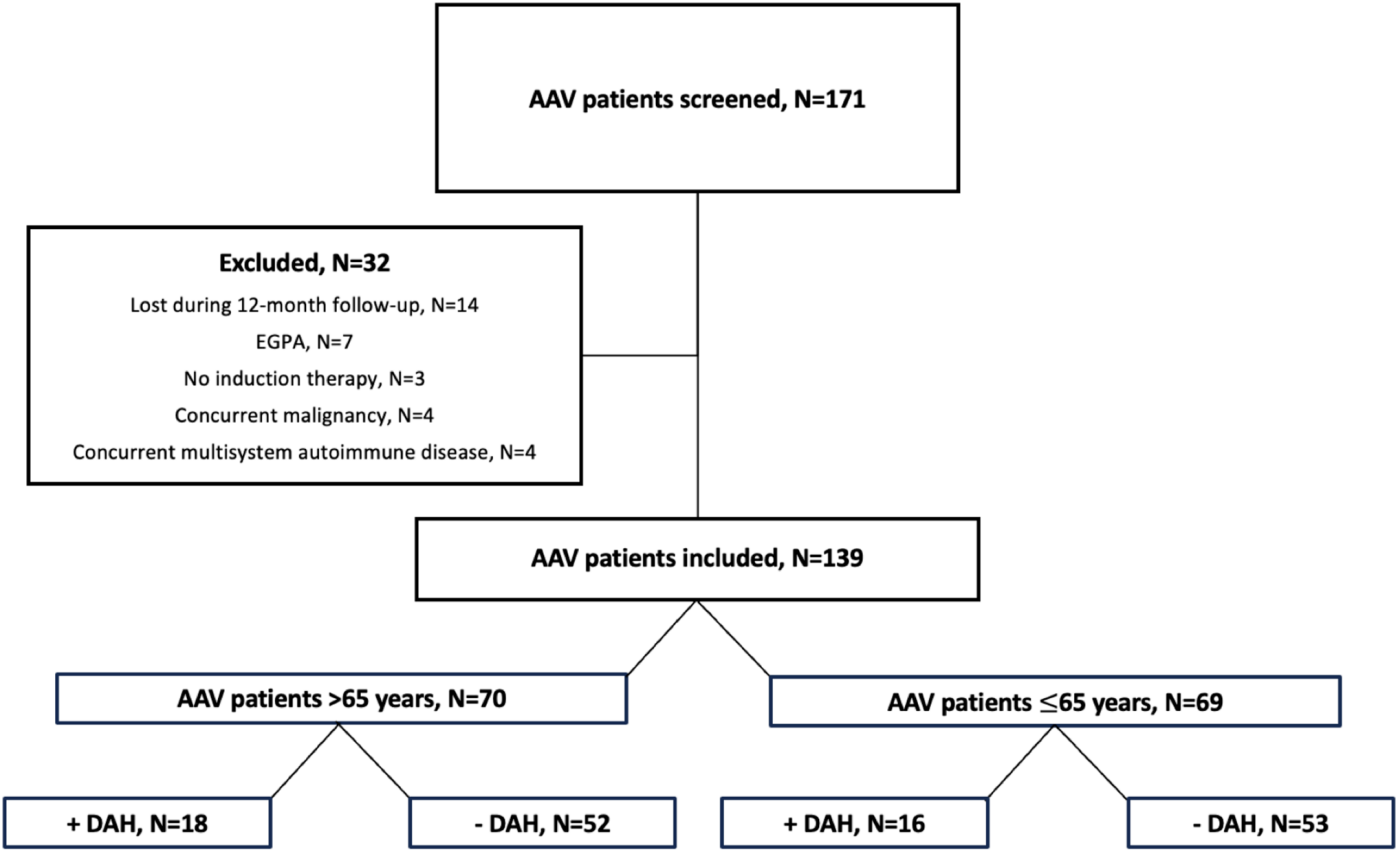
Study cohort. *AAV* ANCA-associated vasculitis, *DAH* diffuse alveolar hemorrhage, *EGPA* eosinophilic granulomatosis with polyangiitis, *N* number

For this retrospective cohort study, we screened 171 patients with new-onset AAV at the Department of Nephrology, University of Heidelberg between 2004 and 2023, of whom 139 were eligible for inclusion (Fig. 1). AAV patients with granulomatosis with polyangiitis (GPA) and microscopic polyangiitis (MPA) were included. The diagnosis was made in accordance with the Chapel Hill disease definitions, including positive ANCA serology and/or histology [14, 15]. The trial was approved by the ethics committee of the University of Heidelberg (ref: S-624/2014). The study was conducted in adherence to the principles outlined in the Declaration of Helsinki.

### Study design and outcome variables

A total of 139 patients were included in our study, 32 patients were excluded due to the exclusion criteria described in detail below (Fig. 1). AAV patients were divided into the following groups: (1) patients ≤ 65 years (N = 69) and (2) patients > 65 years (N = 70), who were defined as “elderly” (Fig. 1). In addition, both AAV patients ≤ 65 years and AAV patients > 65 years were further subdivided into patients with (+) and without (−) DAH (Fig. 1). DAH was confirmed by computed tomography (CT) in combination with clinical symptoms such as hemoptysis. The goal of our study was to investigate the impact of higher age as well as DAH especially in “elderly” AAV patients on different outcome measurements. Primary outcomes included patient survival, relapse-free survival, death by infectious complications, and the incidence of pneumonia. Secondary outcomes were defined as disease activity detected by the Disease Extend Index (DEI) and the Birmingham Vasculitis Activity Score (BVAS) score after 3, 6, and 12 months, respectively, end-stage kidney disease (ESKD), irreversible physical damage estimated by the VDI 1 year after the initial diagnosis, and the incidence and severity of adverse effects associated with immunosuppressive medication. Furthermore, a more detailed analysis of infectious complications was conducted, covering the incidence and frequency of infectious adverse events in general, including urinary tract infections, pneumonia, herpes infections, and sepsis. The pathogen spectrum involved in pneumonia, with a particular focus on opportunistic pathogens, was also examined separately. Opportunistic pathogens included *Aspergillus species, Pneumocystis jirovecii, or the cytomegalovirus.* Notably, patients received trimethroprim/sulfamethoxazole for 3 months during induction therapy according to our internal standard.

Inclusion criteria were newly diagnosed GPA or MPA, a follow-up period of at least 12 months after inclusion or death during this period, induction therapy with cyclophosphamide (CYC) or rituximab (RTX), and a patient age of ≥ 18 years. Patients who did not undergo induction therapy, had a concurrent malignancy or multisystem autoimmune disease at the time of initial manifestation, or had eosinophilic granulomatosis with polyangiitis (EGPA) were excluded from the study.

### Follow-up and the assessment of disease activity

The primary and secondary outcome measurements were evaluated at the five-year follow-up period following the initial AAV diagnosis [16]. A response to treatment was identified by a reduction of 50% or more in the DEI, indicating an improvement in vasculitis symptoms. Remission was defined as the absence of disease activity (DEI and BVAS score of 0) with stable immunosuppressive maintenance therapy for at least 1 month. Relapse was characterized by new or worsening symptoms of systemic vasculitis, accompanied by a DEI or BVAS score of 1 or higher. Refractory disease referred to either no change or an increase in disease activity after 3 months of therapy, or chronic, persistent disease despite optimized immunosuppressive medication [17].

### Statistics

The data are presented as median and interquartile range (IQR), or as number (N) and percentage (%). Continuous variables were evaluated using the nonparametric t-test with Welch's correction, while categorical variables were assessed using the Chi-square test. To estimate the univariate probability of patient survival, relapse-free survival, and death due to infectious complications over a 5-year follow-up period, Kaplan–Meier estimators and the log-rank test were utilized. To study predictors of death by infectious complications, multivariable cox regression analysis was applied by controlling for age, DEI and BVAS score at disease onset [18], DAH, CYC induction dose, plasma exchange therapy, and the glucocorticoid dose after 3 months. The results are presented as odds ratios (ORs) with corresponding 95% confidence interval (CI). Statistical significance was defined as a *P*-value of less than 0.05. The analyses were conducted using GraphPad Prism version 10.2.1 (GraphPad Software, San Diego, CA, USA).

## Results

### Baseline patient characteristics of different subgroup analyses

To investigate different disease- and treatment-related outcomes in elderly AAV patients, the patient population was divided into AAV patients > 65 years and ≤ 65 years and followed-up for 5 years after disease onset (Fig. 1). Detailed patient characteristics of both subgroups are given in Table 1. By definition, elderly AAV patients were significantly older with a median (IQR) age of 71 (69–74) compared to 57 (37–60) (Table 1; *P* < 0.001). The disease activity measured by the DEI and the BVAS score as well as the organ systems involved were comparable between both groups. Patients > 65 years tended to have more comorbidities at disease onset such as condition after myocardial infarction and diabetes type 2 with 10% and 17% versus 3% and 7% in patients ≤ 65 years, respectively (Table 1; *P* = 0.08 and *P* = 0.09). Kidney function in elderly patients was significantly worse with a median (IQR) estimated glomerular filtration rate (eGFR) of 18 (12–49) compared to 30 (23–63) at disease onset in younger patients (Table 1; *P* = 0.03).

**Table 1:** Patient characteristics, outcomes, and complications of AAV patients $\le$ 65 years and > 65 years

	Age $\le$ 65 years (N = 69)	Age > 65 years (N = 70)	*P*
Patient characteristics			
Diagnosis, N (%)			
Granulomatosis with polyangiitis	39 (57)	37 (53)	0.66
Microscopic polyangiitis	30 (43)	33 (47)	0.66
Female, N (%)	33 (48)	39 (56)	0.35
Age at diagnosis, median (IQR), y	57 (37–60)	71 (69–74)	** < 0.001**
Comorbidities at disease onset, N (%)			
Diabetes type 2	5 (7)	12 (17)	0.08
Myocardial infarction	2 (3)	7 (10)	0.09
Heart failure	5 (7)	6 (9)	0.77
Chronic kidney disease (CKD $\ge$ G3a*)	6 (9)	15 (21)	**0.04**
Organ involvement, N (%)			
General symptoms	52 (75)	53 (76)	0.96
Ears, nose, throat	15 (22)	23 (33)	0.14
Kidney	69 (100)	70 (100)	0.99
Diffuse alveolar hemorrhage	16 (23)	18 (26)	0.73
Nerve system	10 (14)	7 (10)	0.42
DEI at disease onset, median (IQR)	7 (3–9)	6 (3–8)	0.34
BVAS at disease onset, median (IQR)	18 (13–24)	18 (12–25)	0.54
eGFR at disease onset, median (IQR), ml/min/1.73m^2^	30 (23–63)	18 (12–49)	**0.03**
Dialysis at disease onset, N (%)	6 (9)	8 (11)	0.59
Outcomes			
Relapse rate, N (%)	26 (38)	30 (43)	0.53
Time to relapse, median (IQR)	26 (15–33)	30 (17–36)	0.64
Refractory disease, N (%)	2 (3)	4 (6)	0.41
Disease activity			
DEI after 3 mo, median (IQR)	0 (0–2)	0 (0–1)	0.50
DEI after 6 mo, median (IQR)	0 (0–1)	0 (0–2)	0.67
DEI after 12 mo, median (IQR)	0 (0–1)	0 (0–1)	0.89
BVAS after 3 mo, median (IQR)	0 (0–4)	0 (0–8)	0.33
BVAS after 6 mo, median (IQR)	0 (0–3)	0 (0–4)	0.84
BVAS after 12 mo, median (IQR)	0 (0–2)	0 (0–4)	0.83
New ESKD, N (%)	7 (10)	8 (11)	0.81
Complications			
Steroid-induced diabetes, N (%)	4 (6)	12 (17)	**0.04**
New-onset arterial hypertension, N (%)	3 (4)	9 (13)	0.07
Malignancy during follow-up, N (%)	4 (6)	4 (6)	0.98
Osteoporosis, N (%)	2 (3)	11 (16)	** < 0.01**
Leukopenia, N (%)	4 (6)	11 (16)	0.06
Infectious complications			
At least 1 infectious complication, N (%)	31 (45)	45 (64)	**0.02**
Infectious episodes per patient, median (IQR)	0 (0–2)	1 (1–4)	**0.01**
Urinary tract infection, N (%)	20 (29)	28 (40)	0.17
Pneumonia, N (%)	9 (13)	26 (37)	** < 0.01**
Opportunistic pneumonia, N (%)	2 (3)	9 (13)	**0.03**
Herpes virus infections, N (%)	7 (10)	12 (17)	0.23
Sepsis, N (%)	3 (4)	10 (14)	**0.04**
VDI after 1 year, median (IQR)	1 (0–1)	1 (1–3)	**0.03**
Overall death during follow-up, N (%)	6 (9)	17 (24)	**0.01**
Death by infection, N (%)	2 (3)	10 (14)	**0.02**

Bold defines statistically sigificant values (*P* < 0.05)

*AAV* ANCA-associated vasculitis, *BVAS* Birmingham Vasculitis Activity Score, *BW* body weight, *CKD* chronic kidney disease (*eGFR < 60 ml/min/1.73^2^), *CYC* cyclophosphamide, *DEI* disease extend index, *ESKD* end-stage kidney disease, *GC* glucocorticoids, *eGFR* estimated glomerular filtration rate, *IIF* indirect immunofluorescence, *IQR* interquartile range, *mo* months, *VDI* vascular damage index

The population of elderly AAV patients was further subdivided into patients with and without DAH. Baseline characteristics including age, gender distribution, and comorbidities at disease onset were comparable between both subgroups (Table 2). The disease activity tended to be higher in patients with DAH as compared to patients without DAH with a median (IQR) DEI of 7 (5–8) and 5 (3–6) (Table 2; *P* = 0.07) and a median (IQR) BVAS score of 21 (15–35) and 16 (11–28) (Table 2; *P* = 0.09). Furthermore, significant differences in kidney function or the incidence of end-stage kidney disease between elderly AAV patients with and without DAH at disease onset.

**Table 2:** Patient characteristics, outcomes, and complications of AAV patients > 65 years with (+) and without (−) DAH

	With (+) DAH (N = 18)	Without (−) DAH (N = 52)	*P*
Patient characteristics			
Diagnosis, N (%)			
Granulomatosis with polyangiitis	7 (39)	30 (58)	0.17
Microscopic polyangiitis	11 61)	22 (42)	0.17
Female, N (%)	9 (50)	30 (57)	0.57
Age at diagnosis, median (IQR), y	74 (69–79)	70 (71–76)	0.21
Comorbidities at disease onset, N (%)			
Diabetes type 2	3 (17)	9 (17)	0.95
Myocardial infarction	2 (11)	5 (10)	0.86
Heart failure	1 (6)	5 (10)	0.60
Chronic kidney disease (CKD $\ge$ G3a*)	2 (11)	13 (25)	0.22
Organ involvement, N (%)			
General symptoms	14 (78)	39 (75)	0.81
Ears, nose, throat	5 (28)	18 (35)	0.59
Kidney	18 (100)	52 (100)	0.99
Nerve system	1 (6)	6 (12)	0.47
DEI at disease onset, median (IQR)	7 (5–8)	5 (3–6)	0.07
BVAS at disease onset, median (IQR)	21 (15–35)	16 (11–28)	0.09
eGFR at disease onset, median (IQR), ml/min/1.73m^2^	18 (10–56)	19 (12–48)	0.86
Dialysis at disease onset, N (%)	2 (11)	6 (12)	0.96
Outcomes			
Relapse rate, N (%)	8 (44)	22 (42)	0.87
Time to relapse, median (IQR)	32 (18–37)	30 (21–35)	0.71
Refractory disease, N (%)	1 (6)	3 (6)	0.97
Disease activity			
DEI after 3 mo, median (IQR)	0 (0–2)	0 (0–1)	0.71
DEI after 6 mo, median (IQR)	0 (0–2)	0 (0–2)	0.38
DEI after 12 mo, median (IQR)	0 (0–1)	0 (0–1)	0.87
BVAS after 3 mo, median (IQR)	0 (0–5)	0 (0–4)	0.88
BVAS after 6 mo, median (IQR)	0 (0–6)	0 (0–3)	0.16
BVAS after 12 mo, median (IQR)	0 (0–2)	0 (0–2)	0.99
New ESKD, N (%)	3 (17)	5 (10)	0.42
Complications			
Steroid-induced diabetes, N (%)	6 (33)	6 (12)	**0.03**
New-onset arterial hypertension, N (%)	4 (22)	5 (10)	0.17
Malignancy during follow-up, N (%)	1 (6)	3 (6)	0.97
Osteoporosis, N (%)	5 (28)	6 (12)	0.10
Leukopenia, N (%)	3 (17)	8 (15)	0.90
Infectious complications			
At least 1 infectious complication, N (%)	15 (83)	30 (58)	0.05
Infectious episodes per patient, median (IQR)	2 (1–5)	1 (0–3)	**0.04**
Urinary tract infection, N (%)	8 (44)	20 (38)	0.66
Pneumonia, N (%)	11 (61)	15 (29)	**0.01**
Opportunistic pneumonia, N (%)	5 (28)	4 (8)	**0.03**
Herpes virus infections, N (%)	3 (10)	9 (17)	0.95
Sepsis, N (%)	6 (33)	4 (8)	< **0.01**
VDI after 1 year, median (IQR)	2 (2–4)	1 (0–2)	** < 0.001**
Overall death during follow-up, N (%)	8 (44)	9 (17)	**0.02**
Death by infection, N (%)	6 (33)	4 (8)	** < 0.01**

Bold defines statistically sigificant values (*P* < 0.05)

*AAV* ANCA-associated vasculitis, *BVAS* Birmingham Vasculitis Activity Score, *BW* body weight, *CKD* chronic kidney disease (*eGFR < 60 ml/min/1.73^2^), *CYC* cyclophosphamide, *DEI* disease extend index, ESKD end-stage kidney disease, *GC* glucocorticoids, *eGFR* estimated glomerular filtration rate, *IIF* indirect immunofluorescence, *IQR* interquartile range, *mo* months, *VDI* vascular damage index

### Disease- and treatment-related outcomes and complications of elderly patients with ANCA-associated vasculitis

The majority of patients in both groups received intravenous CYC as induction therapy combined with a steroid pulse (Supplemental Table S1. Only 3 (4%) patients ≤ 65 and 4 (6%) patients > 65 years had RTX for induction therapy, respectively. The cumulative CYC dose per kilogram body weight was significantly lower in patients > 65 years with a median (IQR) of 34 mg (27–68) compared to 43 mg (28–86) (Supplemental Table S1; *P* = 0.03). For maintenance therapy, immunosuppressive drugs combined with steroids were not different between both groups (Supplemental Table S1). At disease onset, the administered oral steroid dose was significantly lower in elderly patients with a median (IQR) of 48 mg (35–61) than in patients ≤ 65 years with 60 mg (44–70) (Supplemental Table S1; *P* < 0.001). However, 3, 6, and 12 months after disease onset, the steroid dose no longer differed between the two cohorts.

**Fig. 2:**
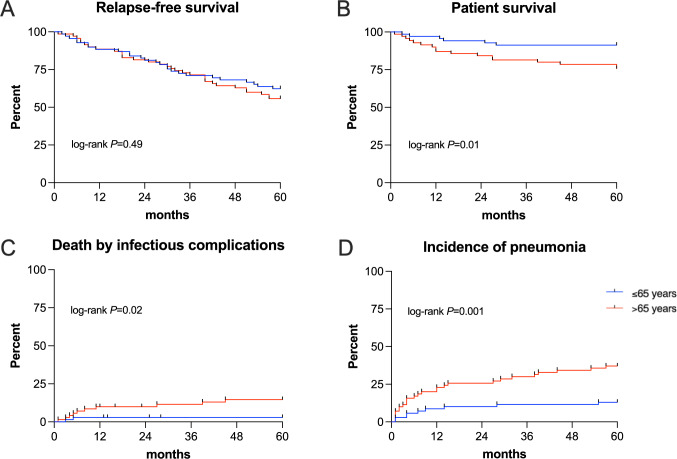
Primary outcomes in AAV patients $\le$ 65 years and > 65 years old. Primary outcomes—relapse-free survival (**A**), patient survival (**B**), death by infectious complications (**C**), and incidence of pneumonia (**D**)—in AAV patients $\le$ 65 years and > 65 years old during a follow-up period of 5 years. To estimate the univariate probability, Kaplan–Meier estimators and the log-rank test were utilized, respectively

Disease-related outcomes such as relapse rate, time to relapse, refractory disease, and disease activity measured by the DEI and the BVAS score 3, 6, and 12 months after disease onset were all comparable between patients > 65 and ≤ 65 years (Table 1). The relapse-free survival as a primary outcome between both groups is shown in Fig. 2A (log-rank *P* = 0.49). The overall survival of patients > 65 years was significantly lower with 17 (24%) compared to 6 (9%) deaths during the 5 years follow-up period (Fig. 2B; log-rank *P* = 0.01). Death by infectious complications occurred more frequently in the elderly cohort with 10 (14%) patients compared to 2 (3%) in the ≤ 65 years group (Fig. 2C; log-rank *P* = 0.02). In addition, the incidence of pneumonia was considerably higher in AAV patients > 65 years with 26 (37%) affected patients than in patients ≤ 65 years with only 9 (13%) patients (Fig. 2D; log-rank *P* = 0.001). Notably, the majority of pneumonias in both subgroups occurred within the first 12 months after disease onset. Opportunistic pathogens (*Aspergillus species, Pneumocystis jirovecii, or the cytomegalovirus)* were significantly more frequently the cause of pneumonia in the > 65 years cohort with 9 (13%) versus 2 (3%) affected patients (Table 1; *P* = 0.03). Three out of 35 patients (9%) with pneumonia had chronic lung disease before the pneumonia occurred. The risk of septic disease courses was also higher in elderly patients (Table 1; *P* = 0.04). Beside infectious complications, the incidences of treatment-related side effects such as steroid-induced diabetes (*P* = 0.04), osteoporosis (*P* = 0.008), new-onset arterial hypertension (*P* = 0.07), and leukopenia (*P* = 0.06) were increased in elderly AAV patients.

### Impact of diffuse alveolar hemorrhage in elderly patients with ANCA-associated vasculitis

To examine the impact of DAH in elderly AAV patients, we further subdivided patients > 65 years into individuals with (N = 18) and without (N = 52) DAH. The CYC or RTX induction dose was not different between both subgroups. However, AAV patients with DAH received significantly more frequently steroid pulse doses (18 (100%) versus 40 (77%), *P* = 0.02; Supplemental Table S2) as well as plasma exchange therapy (8 (44%) versus 5 (10%), *P* = 0.001; Supplemental Table S2) for induction therapy. In addition, with a median (IQR) of 60 mg (49–77) and 20 mg (15–34) the oral steroid dose at disease onset as well as 3 months after induction therapy was significantly higher in AAV patients with DAH as compared to patients without DAH with a median (IQR) of 40 mg (28–58) and 12 mg (8–20), respectively (Supplemental Table S2). Six and 12 months after disease onset, the oral steroid dose was comparable between both groups.

**Fig. 3:**
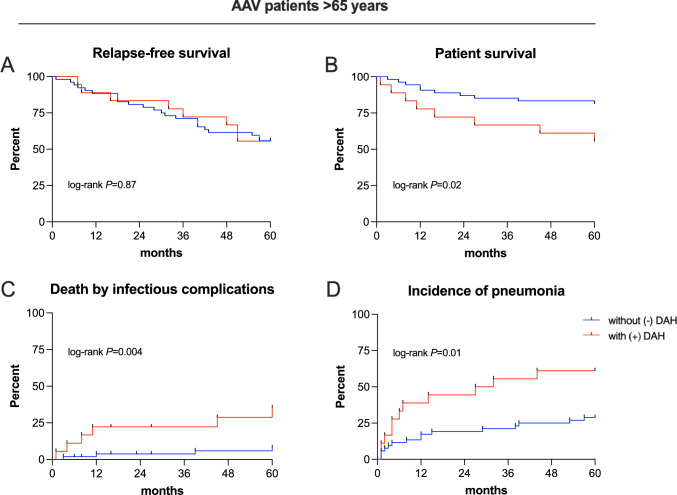
Primary outcomes in elderly AAV patients with (+) and without (−) DAH. Primary outcomes—relapse-free survival (**A**), patient survival (**B**), death by infectious complications (**C**), and incidence of pneumonia (**D**)—in “elderly” AAV patients > 65 years old with (+) and without (−) diffuse alveolar hemorrhage (DAH) during a follow-up period of 5 years. To estimate the univariate probability, Kaplan–Meier estimators and the log-rank test were utilized, respectively

Although the initial DEI and the BVAS score tended to be higher in elderly AAV patients with DAH, disease-related outcomes including the primary outcome relapse-free survival were not significantly different compared to patients without DAH (Table 2 and Fig. 3A). Disease activity as well as renal outcome parameters such as ESKD were comparable during follow-up (Table 2). In contrast, overall patient survival was significantly lower in elderly patients with DAH with 8 (44%) compared to 9 (17%) deaths during the 5 years follow-up (*P* = 0.02; Fig. 3B). Notably, 6 out of 8 fatal outcomes in elderly DAH patients were associated with severe infectious complications, especially pneumonia during the first 6–12 months. Both death by infectious complications [6 (33%) versus 4 (8%), *P* = 0.004] as well as the incidence of pneumonia [11 (61%) versus 15 (29%), *P* = 0.01] were significantly increased in patients with DAH (Fig. 3C + D). Sepsis [6 (33%) versus 4 (8%), *P* = 0.004] and pneumonia with opportunistic pathogens [5 (28%) versus 4 (8%), *P* = 0.03] occurred also more frequently in elderly AAV patients with DAH (Table 2). In addition, with a median (IQR) of 2 (2–4) in elderly patients with and 1 (0–2) in patients without DAH, the VDI 1 year after disease onset was tremendously higher (*P* < 0.001; Table 2). However, the VDI scores irreversible physical damage $\ge$ 3 months after disease onset and is either affected by AAV disease activity or by treatment-related side effects and by implication there is no attribution of cause.

We also performed a detailed characterization of AAV patients ≤ 65 with (N = 16) and without (N = 53) DAH (Supplemental Tables S3 + S4). Except for an increased DEI and BVAS score at disease onset in DAH patients, baseline characteristics and disease-related outcomes were not significantly different between both subgroups (Supplemental Table S3). Comparably to elderly AAV patients with DAH, patients ≤ 65 with DAH more often received plasma exchange and had a significantly higher oral steroid dose at disease onset (Supplemental Table S4). However, whereas younger AAV patients with DAH also had more infectious episodes per patient, severe infectious complications such as pneumonia and sepsis were similarly common and death by infectious complications was consequently not significantly increased as compared to younger AAV patients without DAH (Supplemental Table S3). Overall death during follow-up tended to be higher without being statistically significant (Supplemental Table S3; *P* = 0.10).

### Predictors of death by infectious complications in all patients (N = 139) with ANCA-associated vasculitis

To investigate predictors for death by infectious complications, we performed a multivariable cox regression analysis including all 139 AAV patients enrolled in our study. We controlled for the confounders age, DEI and BVAS score at disease onset, DAH, CYC induction dose, plasma exchange therapy, and the glucocorticoid dose after 3 months. As age was included as a confounding factor in our analysis, we did not subdivide patients into > 65 and ≤ 65 years. Only higher age at disease onset [OR 1.62, CI (1.45–2.12), *P* = 0.01] and higher GC dose after 3 months [OR 1.23, CI (1.05–1.65), *P* = 0.04] independently predicted death by infectious complications (Table 3).

**Table 3:** Predictors of death by infectious complications in all AAV patients (N = 139)

Variable	OR (95% CI)	*P*
Age	1.62 (1.45–2.12)	**0.01**
DEI at disease onset	1.06 (0.92–1.26)	0.39
BVAS at disease onset	1.04 (0.89–1.20)	0.29
Diffuse alveolar hemorrhage	1.20 (0.93–1.45)	0.18
CYC induction dose	1.02 (0.85–1.19)	0.53
Plasma exchange therapy	0.95 (0.84–1.36)	0.47
GC dose after 3 months	1.23 (1.05–1.65)	**0.04**

Bold defines statistically sigificant values (*P* < 0.05)

*AAV* ANCA-associated vasculitis, *BVAS* Birmingham Vasculitis Activity Score, *CI* confidence interval, *CYC* cyclophosphamide, *DEI* disease extend index, *GFR* glomerular filtration rate, *GC* glucocorticoids, *OR* odds ratio

## Discussion

The objective of this study was to examine the influence of DAH as a severe manifestation of AAV in elderly patients on disease-related outcomes and treatment-related complications. In this study, we demonstrate that elderly AAV patients (> 65 years) generally had comparable relapse-free survival and disease activity during the 5-year follow-up compared to younger (≤ 65 years) AAV patients. However, life-threatening infectious complications such as pneumonia with opportunistic pathogens and sepsis occurred significantly more often in older AAV patients, and the number of deaths from infectious complications was consequently higher. Although induction therapy was age-adapted with lower cumulative doses of CYC and GC, elderly AAV patients had GC maintenance doses as high as younger patients 3 months after disease onset. Most importantly, elderly AAV patients with DAH had a mortality rate as high as 44% during the 5-year follow-up with strikingly 33% deaths due to infectious complications while relapse-free survival and disease activity were comparable to elderly AAV patients without DAH. In multivariate analyses, age and GC dose at 3 months were the only predictors of death from infectious complications, whereas this could not be independently demonstrated for DAH.

The mortality and morbidity of elderly patients with AAV are known to be strongly influenced by the prevention of severe infectious complications during the first year after disease onset [19–21]. Rathmann et al. showed that up to 40% of AAV patients experienced a severe infectious complication with older age and higher BVAS score as independent predictive factors [22]. In particular, infections were associated with high rates of organ damage and poorer patient survival [22]. Consistent with our study, Sada et al. observed that older AAV patients with higher GC doses developed more frequent infectious complications during short-term follow-up, while the remission rate and relapses were not significantly increased [23]. These results also confirm our previous study, in which AAV patients with a prednisolone dose of > 7.5 mg had more severe infectious complications 6 months after disease onset without any effect on time to relapse [24]. Rapid tapering of the initial GC dose might be crucial to ensure safe immunosuppressive treatment especially in elderly AAV patients. However, an important study by Weiner et al. showed that elderly AAV patients have a relatively favorable outcome if they survive the first year after disease onset, despite significant treatment-related complications – underscoring the importance and usefulness of appropriately dosed immunosuppression in this vulnerable patient group as well [11]. Therefore, an age-appropriate immunosuppressive therapy appears to be essential to balance the risk between infection and disease progression. However, while an age-adapted induction therapy, e.g. with reduced CYC or steroid doses, is relatively strictly adhered to, our real-life data show that a consistent and early tapering of steroids was often insufficient despite the absence of relapses and low disease activity.

Besides the occurrence of fatal infections, DAH is considered to be an important cause of morbidity and one of the strongest predictors of early mortality in AAV [25, 26]. However, data on DAH in elderly AAV patients is still very limited. In this study we showed that elderly AAV patients with DAH had an enormously high mortality: 8 out of 18 (44%) patients died during follow-up. In addition, the incidence of pneumonia (with and without opportunistic pathogens) leading to death outcomes was significantly higher in older AAV patients with DAH than in patients without DAH. Possible explanations for the increased incidence of pneumonia could be a predisposition of the previously damaged lung, but also intensified immunosuppression. A recent study by Fussner et al. showed that AAV patients with DAH had a high 1-year mortality of 12%, whereas severe infections did not differ by DAH status or treatment [13]. However, the patient age in this post-hoc analysis of the PEXIVAS trial was significantly lower than in our study, with an average age of 61 years [13]. In a subanalysis comparing AAV patients aged ≤ 65 years old with and without DAH, we also observed no significant differences regarding disease-related outcomes and severe infectious complications leading to death. In addition, the VDI of elderly patients with DAH was significantly higher as compared to patients without DAH highlighting the severe burden of disease- and/or treatment-associated damage in this subpopulation. Another study by Caballero-Islas et al. retrospectively compared 57 AAV patients with at least one severe infectious complication with 51 AAV patients without infectious complications during follow-up [27]. They showed that patients with an infection at AAV diagnosis were more likely to have received methylprednisolone boluses and more likely to have lung involvement than patients without infections [27]. In summary, recent evidence suggests that the balance between harm and benefit in elderly AAV patients with DAH is difficult to find, but individualized immunosuppressive treatment that takes into account comorbidities and frailty, disease activity, and risk of infection appears to be critical for both patient survival and preservation of long-term organ function.

This study examined AAV patients enrolled in the last two decades, which is why a large proportion of patients received intravenous CYC induction therapy. However, the landscape of AAV induction therapy has diversified in recent years, and RTX has been shown to be equally effective and have comparable side effects as intravenous CYC [28–30]. Especially for relapsing disease, RTX is the preferred immunosuppressive agent based on data of the RAVE study [31]. A combination of RTX and CYC for induction therapy, each at a reduced dose, has also been shown to be effective and enabled rapid GC tapering, which could be particularly beneficial for older patients [32, 33]. In this and a previous study, we have shown that inadequate GC tapering, rather than CYC induction dose, appears to be associated with severe infectious complications [34]. Therefore, strategies to reduce the cumulative GC burden are essential to reduce severe side effects and consequently improve patient survival and morbidity during long-term follow-up. Avacopan, a selective C5a receptor inhibitor, provides targeted suppression of neutrophil activation and inflammation in AAV patients and has been shown to reduce the need for high-dose GC and improve both the efficacy and safety of long-term maintenance therapy [35, 36]. Older AAV patients in particular could benefit from this combination therapy, and even a complete replacement of GC therapy with Avacopan could be an option, at least in patients with mild disease activity [37].

This study has several limitations. First of all, it is a retrospective study with a relatively small sample size, especially in elderly AAV patients with DAH. However, AAV is by definition an orphan disease and DAH is a rare but severe manifestation, so the number of cases is still relatively large compared to other AAV studies. Another limitation is that most patients received intravenous CYC induction therapy and no conclusions can be drawn in this study for patients with alternative induction regimens such as RTX. In addition, most patients were admitted to a large nephrology center, which may be the reason why our cohort has an exceptionally high disease activity, renal involvement in all patients, and a high number of concomitant diseases. The use of anticoagulants before the onset of the first DAH symptoms was not systematically recorded and can therefore not be ruled out as a cause of DAH. In addition, we did not measure immunoglobulin levels regularly during follow-up which could limit our results since secondary hypogammaglobulinemia significantly increases the risk of infectious complications.

In summary, the new aspect of our current study in the focus on DAH as a relatively rare but serious complication especially in the underrepresented subpopulation of elderly AAV patients. We showed that life-threatening infectious complications with opportunistic pneumonia are common in elderly AAV patients with DAH during the first year after disease onset and higher GC doses during maintenance therapy were an independent predictor of death from infectious complications. Therefore, rapid GC tapering should be carefully re-evaluated to balance the risk between infection and disease progression in this high-risk population.

## Supplementary Information

Below is the link to the electronic supplementary material.

Supplementary file1 (DOCX 29 KB)

## Data Availability

The datasets collected for this manuscript are available from the corresponding author upon reasonable request.
