# Supplementary material for: ANCA-associated vasculitis and the impact of diffuse alveolar hemorrhage in elderly patients: a retrospective cohort study
Source: Rheumatol Int. 2025 Mar 3;45(3):68. doi: 10.1007/s00296-025-05812-8 (PMC11876205; doi:10.1007/s00296-025-05812-8)
Supplement: Supplementary file 1 — Supplementary file1 (DOCX 29 KB) [file 296_2025_5812_MOESM1_ESM.docx]

**Supplemental data**

**Supplemental content**

**Supplemental Table S1.** Immunosuppressive treatment regimens for induction and maintenance therapy in AAV patients $\leq$65 years and >65 years

**Supplemental Table S2.** Immunosuppressive treatment regimens for induction and maintenance therapy in AAV patients >65 years with (+) and without (-) DAH

**Supplemental Table S3.** Patient characteristics, outcomes, and complications of AAV patients $\leq$65 years with (+) and without (-) DAH

**Supplemental Table S4.** Immunosuppressive treatment regimens for induction and maintenance therapy in AAV patients $\leq$65 years with (+) and without (-) DAH

**Supplemental Table S1**

**Immunosuppressive treatment regimens for induction and maintenance therapy in AAV patients** $\boldsymbol{\leq}$**65 years and >65 years**

**Age** $\boldsymbol{\leq}$**65 years Age >65 years *P***

**(N=69) (N=70)**

**Immunosuppressive therapy**

*Induction therapy*

CYC, N (%) 66 (96) 66 (94) 0.98

Cumulative CYC dose, median (IQR), g 3.6 (2.7–5.6) 2.9 (2.5–4.9) **0.02**

Cumulative CYC dose / kg BW, median (IQR), mg 43 (28–86) 34 (27–68) **0.03**

Duration of CYC induction, median (IQR), mo 12 (5–18) 12 (4–16) 0.33

RTX, N (%) 3 (4) 4 (6) 0.98

Cumulative RTX dose, median (IQR), g 3.9 (2.4–5.1) 3.1 (2.0–4.1) 0.62

Duration of RTX induction, median (IQR), mo 2 (2–4) 2 (1–3) 0.78

Steroid pulse therapy, N (%) 51 (74) 58 (83) 0.20

Plasma exchange, N (%) 13 (19) 13 (19) 0.97

*Maintenance therapy*

Azathioprine, N (%) 47 (68) 46 (66) 0.76

Mycophenolic acid, N (%) 7 (10) 12 (17) 0.20

RTX, N (%) 2 (3) 2 (3) 0.99

Exclusively steroids, N (%) 13 (19) 10 (14) 0.47

Steroid dose at disease onset, median (IQR), mg 48 (35–61) 60 (44–70) **<0.001**

Steroid dose after 3 months, median (IQR), mg 16 (9–23) 20 (11–26) 0.28

Steroid dose after 6 months, median (IQR), mg 4 (2–11) 6 (3–14) 0.42

Duration of maintenance therapy, median (IQR), mo 28 (19–32) 33 (22–38) 0.30

AAV, ANCA-associated vasculitis; CYC, cyclophosphamide; DAH, diffuse alveolar hemorrhage; IQR, interquartile range; mo, months; N, number; RTX, Rituximab

**Supplemental Table S2**

**Immunosuppressive treatment regimens for induction and maintenance therapy in AAV patients >65 years with (+) and without (-) DAH**

**With (+) DAH Without (-) DAH *P***

**(N=18) (N=52)**

**Immunosuppressive therapy**

*Induction therapy*

CYC, N (%) 18 (100) 48 (92) 0.23

Cumulative CYC dose, median (IQR), g 3.1 (2.5–5.2) 2.7 (2.2–4.5) 0.40

Cumulative CYC dose / kg BW, median (IQR), mg 36 (28–72) 31 (24–60) 0.53

Duration of CYC induction, median (IQR), mo 14 (6–19) 10 (5–15) 0.37

RTX, N (%) 0 (0) 4 (8) 0.23

Cumulative RTX dose, median (IQR), g - 3.1 (2.0–4.1) -

Duration of RTX induction, median (IQR), mo - 2 (1–3) -

Steroid pulse therapy, N (%) 18 (100) 40 (77) **0.02**

Plasma exchange, N (%) 8 (44) 5 (10) **<0.01**

*Maintenance therapy*

Azathioprine, N (%) 14 (77) 32 (62) 0.21

Mycophenolic acid, N (%) 3 (17) 9 (17) 0.95

RTX, N (%) 0 (0) 2 (4) 0.40

Exclusively steroids, N (%) 1 (6) 9 (17) 0.22

Steroid dose at disease onset, median (IQR), mg 60 (49–77) 40 (28–58) **0.01**

Steroid dose after 3 months, median (IQR), mg 20 (15–34) 12 (8–20) **<0.01**

Steroid dose after 6 months, median (IQR), mg 8 (4–16) 4 (3–9) 0.13

Duration of maintenance therapy, median (IQR), mo 34 (10–60) 33 (10–54) 0.67

AAV, ANCA-associated vasculitis; CYC, cyclophosphamide; DAH, diffuse alveolar hemorrhage; IQR, interquartile range; mo, months; N, number; RTX, Rituximab

**Supplemental Table S3**

**Patient characteristics, outcomes, and complications of AAV patients** $\boldsymbol{\leq}$**65 years with (+) and without (-) DAH**

**With (+) DAH Without (-) DAH *P***

**(N=16) (N=53)**

**Patient characteristics**

*Diagnosis, N (%)*

Granulomatosis with polyangiitis 9 (56) 30 (57) 0.98

Microscopic polyangiitis 7 (44) 23 (43) 0.98

Female, N (%) 9 (56) 24 (45) 0.44

Age at diagnosis, median (IQR), y 57 (29–62) 57 (36–60) 0.70

*Comorbidities at disease onset, N (%)*

Diabetes type 2 0 (0) 3 (6) 0.33

Myocardial infarction 1 (6) 1 (2) 0.36

Heart failure 0 (0) 5 (9) 0.20

Chronic kidney disease (CKD $\geq$ G3a*) 1 (6) 5 (9) 0.69

*Organ involvement, N (%)*

General symptoms 14 (88) 38 (72) 0.20

Ears, nose, throat 3 (19) 12 (23) 0.74

Kidney 16 (100) 53 (100) 0.99

Nerve system 3 (19) 7 (13) 0.58

DEI at disease onset, median (IQR) 8 (5–9) 5 (3–6) **<0.01**

BVAS at disease onset, median (IQR) 21 (16–24) 16 (14–22) **<0.01**

eGFR at disease onset, median (IQR), ml/min/1.73m^2^ 18 (11–35) 30 (28–54) 0.20

Dialysis at disease onset, N (%) 1 (6) 5 (9) 0.69

**Outcomes**

Relapse rate, N (%) 5 (31) 21 (40) 0.54

Time to relapse, median (IQR) 24 (12–30) 28 (16–32) 0.81

Refractory disease, N (%) 1 (6) 1 (2) 0.36

*Disease activity*

DEI after 3 mo, median (IQR) 0 (0–1) 0 (0–2) 0.33

DEI after 6 mo, median (IQR) 0 (0–1) 0 (0–2) 0.65

DEI after 12 mo, median (IQR) 0 (0–1) 0 (0–1) 0.47

BVAS after 3 mo, median (IQR) 0 (0–3) 0 (0–4) 0.34

BVAS after 6 mo, median (IQR) 0 (0–1) 0 (0–4) 0.57

BVAS after 12 mo, median (IQR) 0 (0–1) 0 (0–2) 0.56

New ESKD, N (%) 3 (19) 4 (8) 0.19

**Complications**

Steroid-induced diabetes, N (%) 2 (13) 2 (4) 0.19

New-onset arterial hypertension, N (%) 0 (0) 3 (6) 0.33

Malignancy during follow-up, N (%) 0 (0) 4 (8) 0.26

Osteoporosis, N (%) 0 (0) 2 (4) 0.43

Leukopenia, N (%) 1 (6) 3 (6) 0.93

*Infectious complications*

At least 1 infectious complication, N (%) 11 (69) 20 (38) **0.03**

Infectious episodes per patient, median (IQR) 1 (1–3) 0 (0–1) **0.02**

Urinary tract infection, N (%) 8 (50) 12 (23) **0.04**

Pneumonia, N (%) 3 (19) 6 (11) 0.44

Opportunistic pneumonia, N (%) 1 (6) 1 (2) 0.36

Herpes virus infections, N (%) 3 (19) 4 (8) 0.19

Sepsis, N (%) 0 (0) 3 (6) 0.33

VDI after 1 year, median (IQR) 2 (1–4) 0 (0–1) **<0.01**

Overall death during follow-up, N (%) 3 (19) 3 (6) 0.10

Death by infection, N (%) 1 (6) 1 (2) 0.36

AAV, ANCA-associated vasculitis; BVAS, Birmingham Vasculitis Activity Score; BW, body weight; CKD, chronic kidney disease (*eGFR <60ml/min/1,73^2^); CYC, cyclophosphamide; DEI, disease extend index; DAH, diffuse alveolar hemorrhage; ESKD, end-stage kidney disease; GC, glucocorticoids; eGFR, estimated glomerular filtration rate; IIF, indirect immunofluorescence; IQR, interquartile range; mo, months; N, number; VDI, vascular damage index

**Supplemental Table S4**

**Immunosuppressive treatment regimens for induction and maintenance therapy in AAV patients** $\boldsymbol{\leq}$**65 years with (+) and without (-) DAH**

**With (+) DAH Without (-) DAH *P***

**(N=16) (N=53)**

**Immunosuppressive therapy**

*Induction therapy*

CYC, N (%) 16 (100) 50 (94) 0.33

Cumulative CYC dose, median (IQR), g 3.9 (2.2–7.4) 3.4 (2.8–5.9) 0.12

Cumulative CYC dose / kg BW, median (IQR), mg 42 (23–64) 44 (28–76) 0.24

Duration of CYC induction, median (IQR), mo 13 (6–28) 10 (5–18) 0.31

RTX, N (%) 0 (0) 3 (6) 0.33

Cumulative RTX dose, median (IQR), g - 3.9 (2.4–5.1) -

Duration of RTX induction, median (IQR), mo - 2 (2–4) -

Steroid pulse therapy, N (%) 14 (88) 37 (70) 0.16

Plasma exchange, N (%) 10 (63) 3 (6) **<0.001**

*Maintenance therapy*

Azathioprine, N (%) 13 (81) 34 (64) 0.20

Mycophenolic acid, N (%) 3 (19) 4 (8) 0.19

RTX, N (%) 0 (0) 2 (4) 0.43

Exclusively steroids, N (%) 0 (0) 13 (25) **0.03**

Steroid dose at disease onset, median (IQR), mg 60 (42–81) 60 (40–69) 0.19

Steroid dose after 3 months, median (IQR), mg 20 (14–34) 16 (8–25) **<0.01**

Steroid dose after 6 months, median (IQR), mg 6 (2–14) 6 (2–9) 0.37

Duration of maintenance therapy, median (IQR), mo 30 (16–39) 27 (20–32) 0.84

AAV, ANCA-associated vasculitis; CYC, cyclophosphamide; DAH, diffuse alveolar hemorrhage; IQR, interquartile range; mo, months; N, number; RTX, Rituximab
